# Comparisons of Clinical and Functional Characteristics of Patients with Epiretinal Membrane, Macular Pseudohole, Epiretinal Membrane Foveoschisis, and Lamellar Macular Hole

**DOI:** 10.3390/jcm14227991

**Published:** 2025-11-11

**Authors:** Noriko Kubota, Kazunori Miyata, Yosai Mori, Yuji Nakano, Hitoshi Goto, Fumiki Okamoto

**Affiliations:** 1 Department of Ophthalmology, Nippon Medical School Hospital, 1-1-5 Sendagi, Bunkyo-ku, Tokyo 113-8603, Japanf-okamoto@nms.ac.jp (F.O.); 2 Miyata Eye Hospital, 6-3, Kuraharacho, Miyakonojo 885-0051, Japan; 3 Department of Ophthalmology, Nippon Medical School Tama Nagayama Hospital, 1-7-1, Tama, Tokyo 206-8512, Japan

**Keywords:** epiretinal membrane, macular pseudohole, foveoschisis, lamellar macular hole

## Abstract

**Background/Objectives:** To investigate and compare the clinical characteristics of epiretinal membrane (ERM) and associated diseases: macular pseudohole (MPH), ERM foveoschisis (ERM-FS), and lamellar macular hole (LMH). **Methods:** We retrospectively reviewed of all patients who underwent vitrectomy with at least 6 months follow-up, all eyes were classified into four groups: ERM, MPH, ERM-FS, and LMH. Age, gender, presence of glaucoma, preoperative spherical equivalent, axial length (AL), preoperative and postoperative best-corrected visual acuity (BCVA), metamorphopsia using M-CHARTS^®^, and frequency of overlapping associated diseases were investigated. The association between pre- and postoperative BCVA and these clinical factors was analyzed. **Results:** After enrolling 718 eyes of 662 patients, eyes were classified as ERM (592 eyes), MPH (76 eyes), ERM-FS (63 eyes), and LMH (42 eyes). Overlapping cases included MPH+ERM-FS (17 eyes), ERM-FS+LMH (14 eyes), MPH+LMH (18 eyes), and MPH+ERM-FS+LMH (3 eyes). The AL was significantly longer (*p* < 0.05) in MPH, ERM-FS, and LMH versus ERM. In all groups, BCVA significantly improved after vitrectomy. Although preoperative BCVA was not significantly different among the four groups, postoperative BCVA was significantly worse for LMH versus ERM (*p* < 0.001). Preoperative metamorphopsia was significantly more severe in ERM (0.52 ± 0.51) versus MPH (0.34 ± 0.29) (*p* < 0.05). Postoperative BCVA correlated with preoperative BCVA and age in all groups except LMH. **Conclusions:** Associated diseases often overlap and were more myopic than ERM. Postoperative BCVA was worse in LMH, while preoperative metamorphopsia was severe in ERM. These results highlight the importance of both clinical and functional evaluations in determining surgical indications and predicting visual outcomes.

## 1. Introduction

Recent advances in optical coherence tomography (OCT) over the past decades have enhanced the visualization of the macular microstructure. This has provided medical practitioners with more accurate diagnoses of various macular disorders. Based on the OCT findings, Hubschman et al. reached a consensus on the definition of macular pseudohole (MPH), epiretinal membrane-foveoschisis (ERM-FS), and lamellar macular hole (LMH) [1]. We adopted this standardized OCT-based classification in the present study to ensure diagnostic consistency and comparability with previous research. The standardized definitions and terminology now allow for more accurate research on MPH, ERM-FS, and LMH. Recent studies have also described the clinical characteristics and postoperative outcomes in eyes with these conditions [2,3,4,5,6,7,8,9,10].

However, in clinical practice, these macular disorders often present overlapping OCT features, and their boundaries are not always clearly defined. This overlap makes it difficult to determine the exact diagnosis and optimal timing for surgical intervention. Therefore, clarifying the similarities and differences among these diseases based on clinical characteristics may provide useful insights for clinical decision-making.

Despite now having established diagnostic criteria based on OCT, the clinical understanding of these associated diseases remains limited. Although ERM removal is a common surgical procedure for all of these four disorders, each disorder is presumed to exhibit distinct clinical characteristics. The purpose of the present study was to investigate and compare the clinical characteristics of a large group of patients with ERM, MPH, ERM-FS, and LMH.

## 2. Methods

This retrospective study was approved by the Ethics Committee of Nippon Medical School following the institution’s ethical review procedures [11]. All patients provided written informed consent for their data to be stored in the hospital database and used for research purposes. The study was conducted in accordance with the principles of the Declaration of Helsinki.

### 2.1. Patient Selection and Classification

The medical records of consecutive patients with idiopathic ERM and associated diseases who underwent vitrectomy between April 2020 and December 2023 and who were followed-up for at least 6 months postoperatively were retrospectively reviewed. Based on spectral-domain OCT findings, patients were categorized into four groups: ERM, MPH, ERM-FS, and LMH (Spectralis version 1.8.6.0, Heidelberg Engineering GmbH, Heidelberg, Germany). Exclusion criteria included patients with a previous history of vitreoretinal surgery and ophthalmic disorders, except for mild cataract. Eyes with secondary ERM attributable to retinal vascular disease, uveitis and trauma were also excluded from the study. Clinical details, which included patients’ age, gender, and the presence of glaucoma, were collected from the medical records.

### 2.2. Visual Assessment

Best-corrected visual acuity (BCVA) was measured using a standard Japanese decimal visual acuity chart at a distance of 5 m. For statistical analyses, the decimal values were converted to the logarithm of the minimal angle of resolution (logMAR) units. The spherical equivalent (SE) and axial length (AL) were measured by an autorefractometer (RC-5000, Tomey Corporation, Nagoya, Japan) and ultrasonography (AL-4000, Tomey Corporation, Nagoya, Japan), respectively. Severity of metamorphopsia was evaluated using M-CHARTS^®^ (Inami Co., Tokyo, Japan) under photopic condition. The subjects were measured in both vertical and horizontal meridians, and the mean values were used for data analysis.

### 2.3. Surgical Procedures

Under local anesthesia, 25-gauge pars plana vitrectomy was performed on all patients by two vitreoretinal surgeons (F.O., Y.M.). A small volume (0.1–0.2 mL) of 0.025% brilliant blue G solution was gently applied over the macula, and after adequate staining was achieved, the dye was aspirated. After peeling the ERM, 0.1 mL of the dye was reapplied to the macular region. Subsequently, we then completely peeled the remaining ILM within an area approximately equal to the vertical extent of the optic disc. In cases with EP, ILM was peeled toward the fovea following ERM removal, and then inverted on the fovea. The EP was embedded into the fovea defect with ILM. This technique was applied to all four disease groups. When cataract extraction was required, the lens was removed by phacoemulsification and an intraocular lens was implanted, followed by vitrectomy.

### 2.4. Statistical Analysis

The mean scores and standard deviations were calculated for age, AL, SE, preoperative and postoperative BCVA, and metamorphopsia score. The difference between the pre- and postoperative BCVA was analyzed by Wilcoxon’s signed-rank test. The significance of the differences in age, AL, SE, pre- and postoperative BCVA among the four groups was examined using the Kruskal–Wallis test. Mann–Whitney U test was also used to examine the differences in the findings between the ERM group and each of the other three groups. Spearman’s rank correlation test was used to examine the correlation between pre- and postoperative BCVA, as well as the correlations between pre- or postoperative BCVA and age, AL, and SE. The analysis of the categorical data in cross tables, such as gender and the presence of glaucoma, was performed using Chi-square tests. Results were considered statistically significant when *p* < 0.05. Data analyses were performed using SPSS Statistics (version 29.0, IBM, Armonk, NY, USA).

## 3. Results

**Figure 1:**
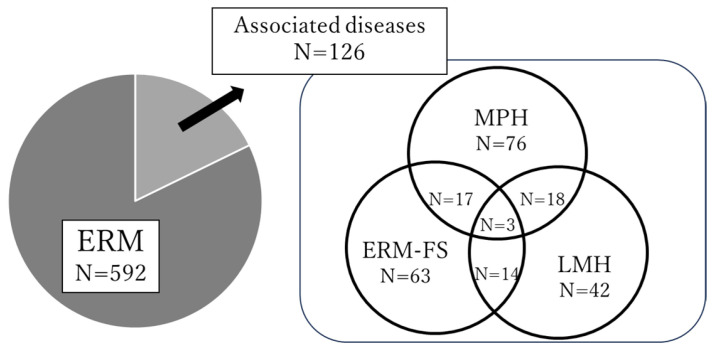
Overlapping frequency of epiretinal membrane (ERM) and associated diseases. MPH = macular pseudohole, ERM-FS = ERM-foveoschisis, LMH = lamellar macular hole. A total of 126 eyes were classified as associated diseases, including overlapping cases: MPH+ERM-FS (17 eyes), ERM-FS+LMH (14 eyes), MPH+LMH (18 eyes), and MPH+ERM-FS+LMH (3 eyes).

A total of 718 eyes from 662 patients (340 men and 322 women) were included in this study, and 639 eyes underwent combined cataract surgery and pars plana vitrectomy. Figure 1 shows the classification results and the frequency of overlapping. Of the 718 eyes with ERM, 592 eyes were typical ERM and 126 eyes had associated diseases, which included 76 eyes with MPH, 63 eyes with ERM-FS, and 42 eyes with LMH. Figure 1 additionally shows that 38 (50%) of the MPH eyes, 34 (54%) of the ERM-FS eyes, and 35 (83%) of the LMH eyes met two or three of the criteria for associated diseases.

Table 1 presents the clinical characteristics of patients with four diseases. In these patients, no significant differences were noted between the groups for the age or gender ratio, and for the glaucoma prevalences, which were 10.1%, 10.5%, 12.7%, and 14.3% for the ERM, MPH, ERM-FS, and LMH, respectively. Compared to the ERM, the mean AL was significantly longer in the MPH, ERM-FS, and LMH. The preoperative metamorphopsia score was significantly higher in the ERM versus the MPH. Although preoperative BCVA was not significantly different among the four groups, the postoperative BCVA was significantly worse in the LMH group versus the ERM group.

**Figure 2:**
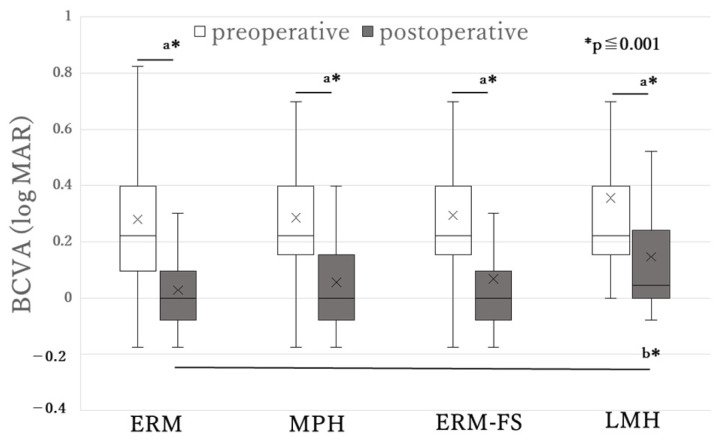
Best-corrected visual acuity (BCVA) before and after surgery in epiretinal membrane (ERM) and associated diseases. MPH = macular pseudohole, ERM-FS = ERM-foveoschisis, LMH = lamellar macular hole, BCVA = best-corrected visual acuity. a: Significantly different in the Wilcoxon’s signed-rank test; b: Significantly different in the Kruskal–Wallis test. The line and cross mark in each box represent the median and mean, respectively. Postoperative BCVA was significantly worse in the LMH versus the ERM, although the baseline BCVA did not significantly differ among the four groups.

Figure 2 shows the changes in the BCVA across the four groups. In all groups, BCVA significantly improved after vitrectomy (*p* < 0.001 in each group). The baseline BCVA did not significantly differ among the four groups. Postoperative BCVA improvement was observed in all groups, with a significant difference found between the ERM and LMH groups.

Table 2 presents the correlations between the preoperative BCVA and the other factors in each group. There was a positive correlation between the preoperative BCVA and age in ERM, which indicates that older patients had a worse preoperative BCVA. In the ERM-FS, a positive correlation was found between the preoperative BCVA and AL, which suggests that longer ALs were associated with worse preoperative BCVA. There was a negative correlation between the preoperative BCVA and the metamorphopsia score in the MPH, which indicates that higher metamorphopsia scores were associated with better preoperative BCVA.

Table 3 shows the correlations between the postoperative BCVA and the other factors in each group. Postoperative BCVA was significantly associated with the preoperative BCVA in all groups. For the ERM, a positive association was found between postoperative BCVA and age, whereas AL was negatively associated with postoperative BCVA. In the MPH and ERM-FS groups, the postoperative BCVA was positively correlated with age. No significant correlations were found between the postoperative BCVA and the other factors in the LMH.

## 4. Discussion

The aim of the consensus on the definitions of MPH, ERM-FS, and LMH that was reached in 2020 was to address the frequent confusion that was often seen in the literature regarding these conditions [2]. However, no studies have compared the clinical differences among ERM and its associated disorders. Despite these clearer distinctions, our study identified a relatively high frequency of overlap among the three associated diseases, with many cases presenting two or more mandatory OCT criteria. Although the present definitions are based on B-scan OCT images, some of these cases exhibited different features depending on the location of the fovea, thereby making the classification difficult. Sasaki et al. [9]. have also reported finding 19 eyes (4.4%) out of 432 eyes with ERM-related disorders that were unclassifiable or had features of two different subtypes. In addition, Matoba et al. [10] reported that 43 eyes (34.1%) out of 126 eyes with associated diseases were mixed types that satisfied at least two of the diagnostic criteria. Therefore, not only B-scan OCT images but also radial OCT scans need to be conducted to make an accurate diagnosis of these associated diseases.

In the present study, a notably high proportion (69%) of the LMH cases exhibited features of two or three associated diseases. Although this was a retrospective study, some eyes showed morphological features suggestive of a sequential relationship among ERM, MPH, ERM-FS, and LMH. When analyzing LMH morphology, findings such as a foveal cavity with poorly defined borders and a visible reduction in foveal tissue should be considered, we found that these were frequently observed in the eyes that were initially detected as ERM, MPH, or ERM-FS. Unlike for the other conditions, LMH does not require the presence of ERM for its mandatory criteria. However, the coexistence of ERM or EP in LMH cases suggests that these components may play a role in its structural development. Thus, LMH may present with features of both ERM and epiretinal proliferation (EP), which are suggested to consist of various cell types, including hyalocytes, fibroblasts, myofibroblasts, and retinal glia cells and Müller cells, respectively [12,13,14,15]. Therefore, the presence of ERM and EP may play a crucial role in the morphological changes that occur in the macula.

In cases where we tracked the macular morphological changes, observing transitions from ERM, MPH or ERM-FS to LMH, we found that the loss of tissue in the fovea, which is a characteristic of LMH, often preceded the appearance of the EP. Thus, this sequence suggests that EP may be produced as a compensatory response to tissue loss as Müller glia cells, and which could potentially be the origin of EP. Moreover, as Müller glia cells are a primary glial component of the retina, they may play a key role in tissue regeneration [16]. Matoba et al. [15] suggested that retinal tissue loss is the primary pathophysiology of LMH and that it can be treated via restoration of the foveal structure by EP embedding (or sparing) surgery.

The associated diseases, MPH, ERM-FS, and LMH, were found to be associated with greater myopic than ERM. A previous study additionally reported that the preoperative refraction in LMH and ERM-FS were −3.4 ± 5.6 D and −3.4 ± 0.6 D, respectively [6]. It has been suggested that MPH, ERM-FS, and LMH are among the macular disorders associated with myopia, along with conditions such as myopic traction maculopathy, foveoschisis, maculoschisis, retinoschisis, and full-thickness macular holes [17].

Although there was no significant difference in the preoperative BCVA among the groups, the postoperative BCVA was significantly worse in the LMH group compared with the ERM. However, no significant differences were observed between each of the other three diseases. It has also been reported that preoperative BCVA did not differ significantly between ERM-FS and LMH [18], or among LMH, ERM-FS, and MPH [3]. The preoperative BCVA is one of the most important factors in determining the need for surgery, and thus, the preoperative BCVA might not significantly differ between diseases. Pertile et al. [18] reported finding no significant differences between ERM-FS and LMH in the BCVA after 24 months of surgery, although the BCVA improvement was significantly earlier in eyes with ERM-FS versus LMH. In addition, Mohammed et al. [19] reported that vitrectomy significantly improved BCVA in MPH and ERM-FS but not in LMH, and that eyes without ellipsoid zone disruption showed significantly better postoperative visual improvement. These findings suggest that timely vitrectomy before the onset of degenerative changes, such as disruption of the ellipsoid zone, may help preserve postoperative visual function.

The preoperative metamorphopsia score was significantly higher in the ERM group versus that observed in the MPH group. We have also previously compared the metamorphopsia score among vitreoretinal disorders and found that the preoperative metamorphopsia score in ERM was significantly higher than that observed in diabetic macular edema [20]. This suggests that ERM is a representative disorder that exhibits metamorphopsia. Interestingly, preoperative BCVA was not correlated with the metamorphopsia score in the ERM group, whereas a better preoperative BCVA was correlated with a higher metamorphopsia score in the MPH group. This result suggests that even patients with relatively good visual acuity may be highly aware of metamorphopsia, which suggests that visual acuity alone is insufficient for evaluating the overall visual function. As previously reported in patients with various vitreoretinal disorders, it is essential to consider various examinations, including the assessment of metamorphopsia, as this can significantly affect the vision-related quality of life [21,22,23].

We also examined the relationship between the pre- and postoperative BCVA and clinical findings. In the ERM group, both preoperative and postoperative BCVA were significantly associated with age, with older patients exhibiting a worse BCVA. In the MPH and ERM-FS group, postoperative BCVA was significantly associated with age. Other studies have also reported that older patients with ERM had worse postoperative BCVA [24,25]. These findings that patients with not only ERM but also MPH and ERM-FS may benefit from undergoing surgery at a relatively younger age to achieve better visual acuity outcomes.

In all groups, the postoperative BCVA was significantly associated with the preoperative BCVA. Previous reports have also demonstrated that better a preoperative BCVA may lead to a better postoperative BCVA [26,27]. Not only the presence of ERM itself but also photoreceptor damage caused by tractional forces of ERM can result in poor visual acuity. As mentioned earlier, the three associated diseases may progress from ERM, indicating advanced tractional and degenerative changes. Therefore, surgery needs to be performed before these irreversible changes occur in the retina.

In ERM-FS, the longer AL was significantly associated with a worse preoperative BCVA, while no significant association was found between the postoperative BCVA and AL. As previously discussed, AL was significantly longer in the MPH, ERM-FS, and LMH groups compared to the ERM group. Although no significant difference was found among these three associated diseases, preoperative BCVA was associated with AL only in the ERM-FS group. The eyes with ERM-FS and a long AL may also benefit from surgery, even if the preoperative BCVA is low. In contrast, in the ERM group, a longer AL was associated with better postoperative BCVA. This may be partly explained by the fact that eyes with longer AL tend to belong to younger patients, in whom posterior vitreous detachment has often already occurred, thereby reducing the intraoperative tractional stress on the retina and facilitating better visual recovery after surgery.

Several factors should be acknowledged as limitations: the retrospective design, the short duration of follow-up, and the emphasis solely on clinical findings. Future studies should aim to establish optimal treatment strategies for each type of disease based on both the clinical characteristics and OCT findings. Although the surgical procedures for these diseases are similar, and which involve peeling of the ERM and ILM, the best surgical approach for each disease may differ.

## 5. Conclusions

We analyzed the clinical characteristics of a relatively large number of patients with ERM and associated diseases, and then compared the differences among these conditions. This study newly demonstrated a high frequency of overlap and distinct clinical features among the associated diseases. These associated diseases being more myopic than ERM. Preoperative metamorphopsia was severe in ERM. Postoperative BCVA was worse in the LMH group versus the ERM group and was worse in older patients in all groups except for the LMH group. Additionally, postoperative BCVA was significantly associated with the preoperative BCVA in all groups. These findings indicate that age and preoperative visual function are important factors influencing surgical outcomes. Therefore, timely surgical intervention should be considered before substantial visual deterioration occurs, particularly in elderly patients or those presenting with already reduced BCVA.

## Figures and Tables

**Table 1:** Clinical characteristics of epiretinal membrane (ERM) and associated diseases.

	ERM	MPH	ERM-FS	LMH
Number of eyes	592	76	63	42
Age, mean ± SD, years	71.7 ± 8.2	71.1 ± 7.8	69.6 ± 8.8	71.3 ± 8.2
Gender, Men/Women	315/277	37/39	28/35	21/21
Prevalences of glaucoma (%)	60 (10.1%)	8 (10.5%)	8 (12.7%)	6 (14.3%)
AL, mm	24.0 ± 1.38	24.8 ± 2.01 *	24.8 ± 2.00 *	24.9 ±1.88 *
SE, diopter	−0.94 ± 3.02	−2.41 ± 4.87	−2.32 ± 4.93	−2.59 ± 4.57
Preoperative BCVA	0.29 ± 0.26	0.29 ± 0.25	0.29 ± 0.30	0.36 ± 0.33
Postoperative BCVA	0.03 ± 0.19	0.06 ± 0.17	0.07 ± 0.24	0.15 ± 0.26 ^†^
Preoperative metamorphopsia score	0.52 ± 0.51 *	0.34 ± 0.29 *	0.36 ± 0.30	0.46 ± 0.38

MPH = macular pseudohole, ERM-FS = ERM-foveoschisis, LMH = lamellar macular hole, AL = axial length, SE = spherical equivalent, BCVA = best-corrected visual acuity. Values are presented as the mean ± standard deviation. Significantly different from the other groups. (AL: *p* < 0.05 ERM vs. MPH, ERM-FS, LMH, Postoperative BCVA: *p* = 0.001 ERM vs. LMH, Preoperative metamorphopsia score: *p* < 0.05 ERM vs. MPH) (* *p* < 0.05, ^†^ *p* = 0.001, Kruskal–Wallis test).

**Table 2:** Correlations between preoperative best-corrected visual acuity (BCVA) and the other factors in epiretinal membrane (ERM) and associated diseases.

	ERM		MPH		ERM-FS		LMH	
	r	*p* Value	r	*p* Value	r	*p* Value	r	*p* Value
Age	0.113	<0.01 *	0.211	0.071	−0.159	0.212	−0.198	0.208
AL	−0.033	0.455	0.086	0.481	0.281	<0.05 *	0.069	0.68
SE	−0.042	0.318	−0.134	0.254	−0.146	0.253	0.05	0.755
Metamorphopsia score	0.012	0.848	−0.353	<0.05 *	0.145	0.437	0.279	0.197

MPH = macular pseudohole, ERM-FS = ERM-foveoschisis, LMH = lamellar macular hole, AL = axial length, SE = spherical equivalent. * Significantly correlated with preoperative BCVA (Spearman’s rank correlation test).

**Table 3:** Correlations between postoperative best-corrected visual acuity (BCVA) and the other factors in epiretinal membrane (ERM) and associated diseases.

	ERM		MPH		ERM-FS		LMH	
	r	*p* Value	r	*p* Value	r	*p* Value	r	*p* Value
Preoperative BCVA	0.423	<0.001 *	0.412	<0.001 *	0.339	0.006 *	0.522	<0.001 *
Age	0.31	<0.001 *	0.452	<0.001 *	0.256	<0.05 *	0.218	0.171
AL	−0.142	0.001 *	−0.096	0.427	0.136	0.311	−0.282	0.091
SE	0.079	0.055	0.023	0.849	−0.003	0.980	0.188	0.238
Metamorphopsia score	−0.089	0.145	0.05	0.774	0.09	0.631	0.085	0.699

MPH = macular pseudohole, ERM-FS = ERM-foveoschisis, LMH = lamellar macular hole, AL = axial length, SE = spherical equivalent. * Significantly correlated with postoperative BCVA (Spearman’s rank correlation test).

## Data Availability

Data can be made available from the corresponding author at oishinoriko@nms.ac.jp on a reasonable request.
